# Supplementary material for: Evidence for functional pre-coupled complexes of receptor heteromers and adenylyl cyclase
Source: Nat Commun. 2018 Mar 28;9:1242. doi: 10.1038/s41467-018-03522-3 (PMC5871782; doi:10.1038/s41467-018-03522-3)
Supplement: Supplementary file 1 — Supplementary Information [file 41467_2018_3522_MOESM1_ESM.pdf]

Supplementary Information

Evidence for functional pre-coupled complexes of receptor heteromers and adenylyl cyclase

Navarro *et al.*

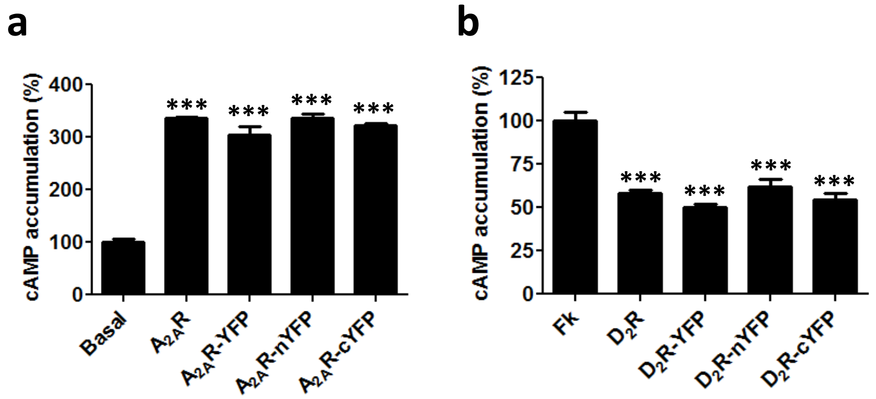

Supplementary Fig. 1 Functionality of fusion proteins. **a.** cAMP production in HEK-293T cells transfected with $A_{2A}R$, $A_{2A}R$-YFP, $A_{2A}R$-nYFP or $A_{2A}R$-cYFP cDNA (0.5 µg), stimulated with CGS 21680 (100 nM). **b.** cAMP production in HEK-293T cells transfected with D_2R, D_2R-YFP, D_2R-nYFP or D_2R-cYFP cDNA (0.75 µg), stimulated with forskolin (Fk; 0.5 µM) in the presence of quinpirole (1 µM). Values (in means ± SEM) are expressed as percentage of cAMP accumulation in non-treated cells (**a**) or as percentage of Fk-treated cells (n = 8, with triplicates) (**b**). ***: $p < 0.001$ as compared as compared to basal values or to Fk; no significant differences were observed with the effects of the different fusion proteins versus the respective non-fused receptor (one-way ANOVA followed by Tukey's multiple comparison tests).

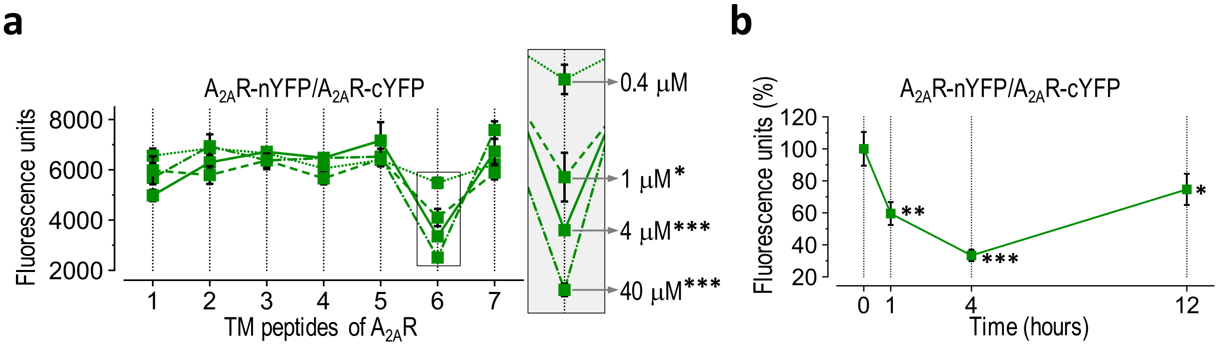

Supplementary Fig. 2 Concentration- and time-dependent destabilizing effect of $A_{2A}R$ TM peptides. BiFC experiments in HEK-293T cells transfected with $A_{2A}R$-nYFP and $A_{2A}R$-cYFP cDNA (0.5 μg) and treated: (**a**) 4 h with diffferent concentrations (0.4, 1, 4 and 40 μM) of TM peptides of $A_{2A}R$ (numbered 1-7) or (**b**) with medium with $A_{2A}R$ TM6 peptide (4 μM) during different incubation times (0, 1, 4 and 12 h). In **a**, magnification of values corresponding to $A_{2A}R$ TM6 peptide is shown. Fluorescence (in means ± S.E.M.) was detected at 530 nm and values are expressed as fluorescence arbitrary units (n = 9, with triplicates); *, ** and *** represent significantly lower values as compared to cells treated with the 0.4 μM concentration (**a**) or with the 0-hour incubation time (**b**) ($p < 0.05$, $p < 0.01$ and $p < 0.001$, respectively; one-way ANOVA followed by Dunnett's multiple comparison tests).

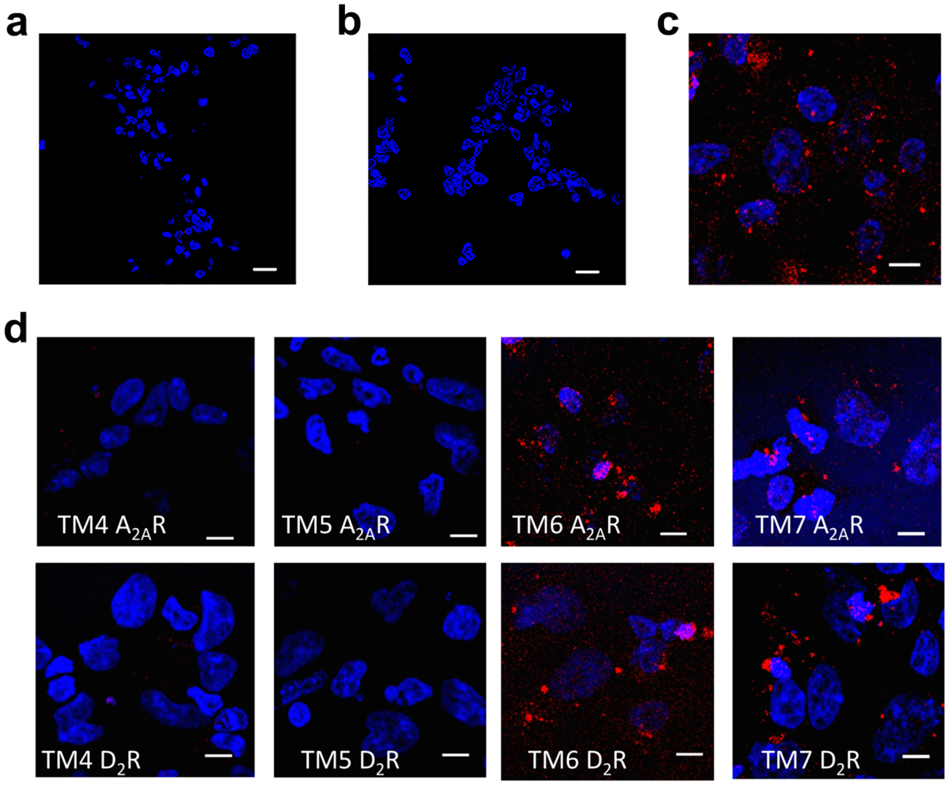

Supplementary Fig. 3 Destabilizing effect of TM peptides on $A_{2A}R$-D_2R heteromerization in transfected cells. Proximity Ligation assay (PLA) in HEK-293T cells transfected with 0.4 µg of $A_{2A}R$ cDNA (**a**), 0.5 µg of D_2R cDNA (**b**) or both (**c** and **d**), treated for 4 h with medium (**a-c**) or with 4 µM of indicated TM peptides of $A_{2A}R$ or D_2R (**d**); confocal microscopy images (superimposed sections) shows $A_{2A}R$-D_2R heteromers as red spots; cell nuclei were stained with DAPI (blue); scale bars: 20 µm.

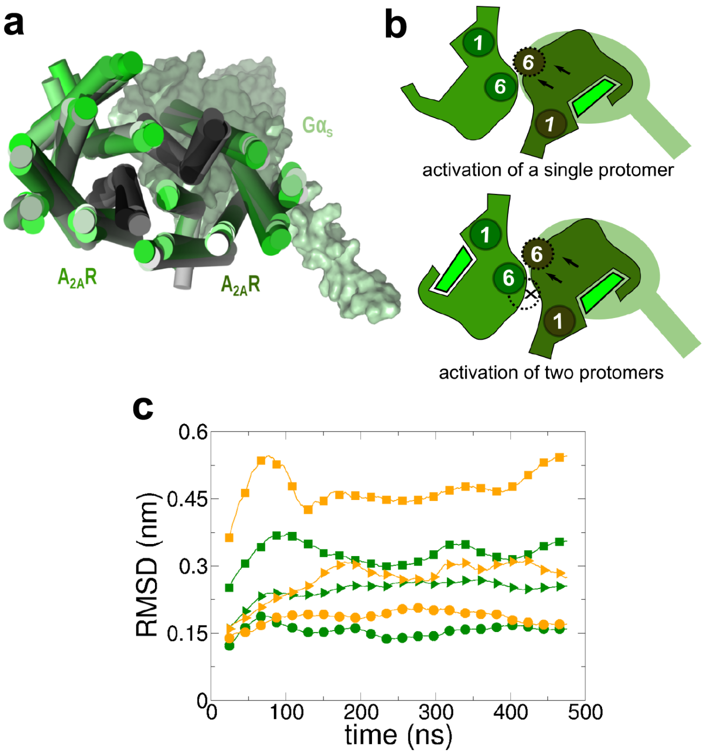

Supplementary Fig. 4 Construction of computational models of the $A_{2A}R$ homodimer in complex with Gs and the D_2R homodimer in complex with Gi. **a** and **b.** Agonist binding opens an intracellular cavity, required for the binding of the C-terminal α5 helix of the G-protein, mainly through the outward movement of TM 6. Thus, the structures for $A_{2A}R$ and D_2R homodimers were modeled with TM 6 in the inactive closed conformation for the unliganded protomer and with TM 6 in the active open conformation for the G protein-bound protomer. TM 6 of the unliganded protomer interacts with TM 6 of the G protein-bound protomer (see cartoon models). It is important to note that, in these models, simultaneous outward movements of TM 6 in the homodimer is not feasible due to a steric clash between active open conformation of both TM 6. Likewise, simultaneous binding of two G proteins to the homodimer would not be possible due to a steric clash between both bulky G proteins. The structures of the $A_{2A}R$ (inactive and Gs-bound "active") and D_2R (inactive and Gi-bound "active") homodimers were constructed using molecular dynamics (MD) simulations due to the absence of crystal structures of oligomers using exclusively the TM 6 interface. In (**a**), representation of 6 evenly spaced snapshots extracted from 500 ns explicit membrane MD trajectories of the $A_{2A}R$ homodimer in complex with Gαs; $A_{2A}R$ homodimers are shown as cylinders in white-to-green color gradient in relation to simulation time; Gαs is shown as a surface, and lipid and solvent molecules are not shown for clarity; similar results were obtained for the D_2R homodimer (data not shown). These results indicate that the possible rotation of "active" protomers relative to inactive protomers through the TM 6 interface is highly limited, since the accessible area of TM 6 is small. In (**c**), time-evolution of the root-mean-square deviations (rmsd) on protein α-carbons in the MD simulations of the $A_{2A}R$ homodimer in complex with Gαs (green) and the D_2R homodimer in complex with Gαi (orange) computed for the whole system (squares), for the homodimers (triangles), and for the residues forming TM 6 (circles). Simulations were performed with the GROMACS 5.0.6 simulation package[1], using the AMBER99SB force field and Berger parameters for POPC lipids. This procedure has been previously validated[2]. The systems consisted on rectangular boxes containing a lipid bilayer (~380 molecules of POPC), explicit solvent (~44,000 water molecules) and a 0.15 M concentration of Na^+ and Cl^-.

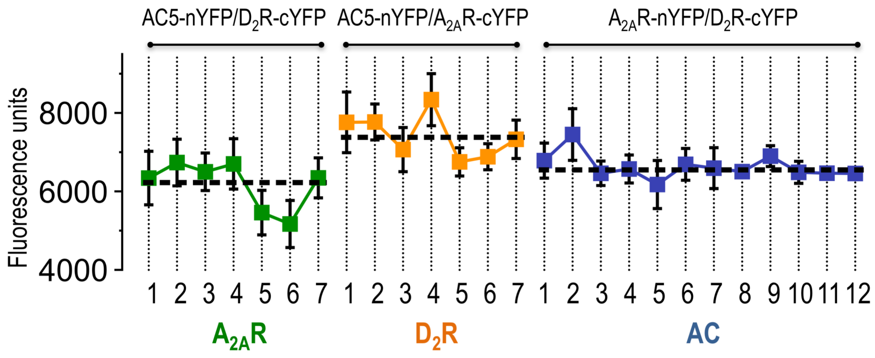

Supplementary Fig. 5 Negative controls for the disrupting effect of TMs peptides. BiFC experiments in HEK-293T cells transfected with AC5-nYFP (0.75 µg) and D_2R-cYFP (0.75 µg) and cDNA (AC5-nYFP/D_2R-cYFP), AC5-nYFP (0.75 µg) and $A_{2A}R$-cYFP (0.5 µg) cDNA (AC5-nYFP/$A_{2A}R$-cYFP) or $A_{2A}R$-nYFP (0.6 µg) and D_2R-cYFP (0.6 µg) cDNA ($A_{2A}R$-nYFP/D_2R-cYFP). Cells were treated for 4 h with medium (control, broken lines), TM peptides of $A_{2A}R$ (4 µM; numbered 1-7, green squares), TM peptides of D_2R (4 µM; numbered 1-7, orange squares) or TM peptides of AC5 (4 µM; numbered 1-12, blue squares). Fluorescence was detected at 530 nm and values (in means ± S.E.M) are expressed as fluorescence arbitrary units (n = 8, with triplicates); no significant differences were observed between any of the peptide-treated groups versus the respective control (one-way ANOVA followed by Dunnett's multiple comparison tests).

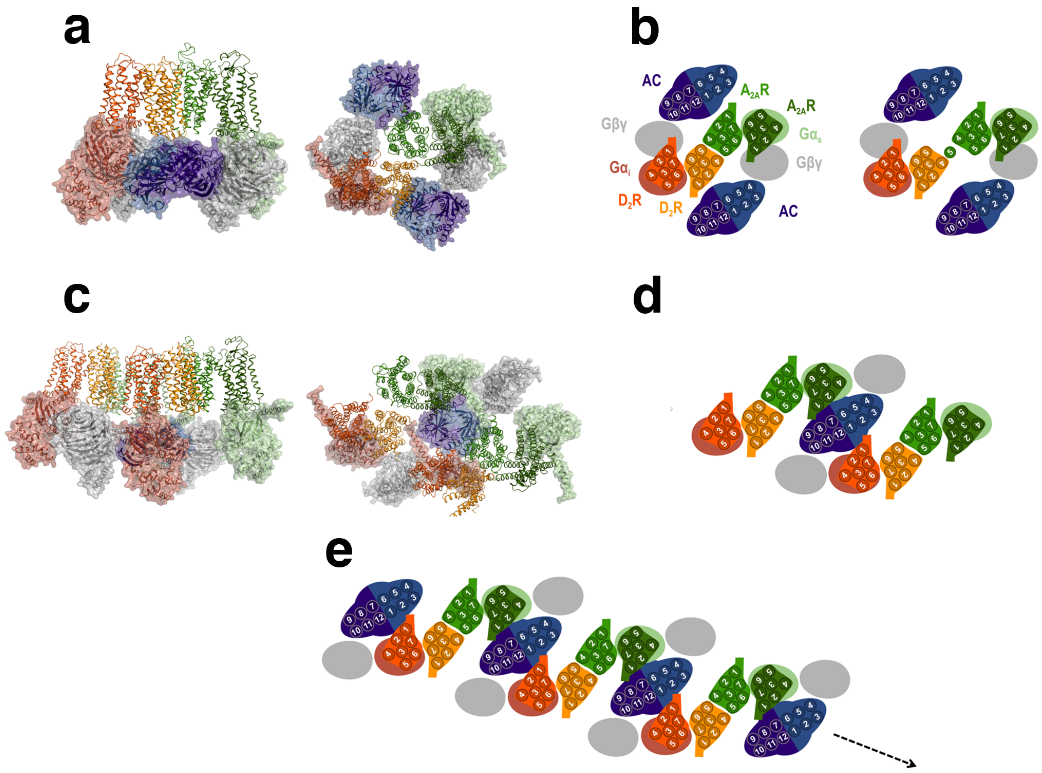

Supplementary Fig. 6 Construction of a computational model of the $A_{2A}R$-D_2R heterotetramer in complex with Gs, Gi and AC5 in the absence and presence of agonists. **a.** Computational molecular model of the $A_{2A}R$-D_2R heterotetramer in complex with Gs, Gi, and the C1 and C2 domains of AC5 in the absence of agonists viewed from the membrane (left image) and from the extracellular side (right image). The $A_{2A}R$-D_2R heterotetramer was built by superimposing the modeled $A_{2A}R$ homodimer in complex with Gs and the D_2R homodimer in complex with Gi (Supplementary Fig. 2) into the oligomeric structure of the β_1-adrenoceptor (PDB code 4GPO) that contains the proposed TMs 4/5 interface between protomers[3]. The relative orientation of pre-coupled G proteins (Gs and Gi) relative to their bound receptors was modeled based on the crystal structure of β_2-adrenoceptor in complex with Gs (PDB code 3SN6)[4]. The topology of AC consists of a variable cytoplasmic N-terminus and two large cytoplasmic C1 and C2 domains, separated by two membrane-spanning M1 and M2 domains, each comprised by six TM helices. The TMs of the M1 and M2 domains of AC are not included in the model due to the absence of crystal structures of these domains or close protein templates suitable for accurate homology modeling. Nevertheless, the results with interfering peptides indicate a direct interaction between TM domains of AC5 and the $A_{2A}R$-D_2R heterotetramer. Moreover, despite there is not structural information regarding the relative localization of the C1 and C2 domains of AC5 relative to the G proteins in the inactive state, the existence of intermolecular interactions of Gsα and Gβγ subunits with the cytoplasmic N-terminus of AC5 have been reported[5,6]. Thus, the C1 and C2 domains (PDB id 1CUL)[7] were positioned between the $A_{2A}R$-D_2R heterotetramer and the G protein to facilitate these transmembrane and intracellular interactions. **b.** Scheme of the molecular model shown in A (viewed from the extracellular side); the scheme at the right side shows the effect of TM5 of $A_{2A}R$ interfering with $A_{2A}R$-D_2R heteromerization and with TM-dependent oligomerization of AC5 with $A_{2A}R$ and D_2R; NT-dependent pre-coupling of AC5 with Gβγ subunits keeps each homodimer separately functional. **c.** Computational molecular model of the $A_{2A}R$-D_2R heterotetramer in complex with Gs, Gi, and the C1 and

C2 domains of AC5 in the presence of agonists viewed from the membrane (left image) and from the extracellular side (right image). **d.** Scheme of the molecular model shown in C (viewed from the extracellular side). The crystal structure of the C1 and C2 domains of AC5 in complex with Gsα (PDB id 1CUL)[7] was used to model the simultaneous binding of Gsα and Giα to a single AC5. The structure of the $A_{2A}R$-D_2R heterotetramer is as in panels A and B. Gβγ subunits were arbitrarily positioned to facilitate the interaction with the cytoplasmic N-terminus of AC5[5]. The same color code applies to schematical and tridimentional models. **e.** The experimental data suggests the possible formation of zig-zagged arranged high-order oligomeric structures, constituted by alternative links of $A_{2A}R$-D_2R heterotetramers and AC5 molecules. The black arrow indicates the direction of possible further expansion.

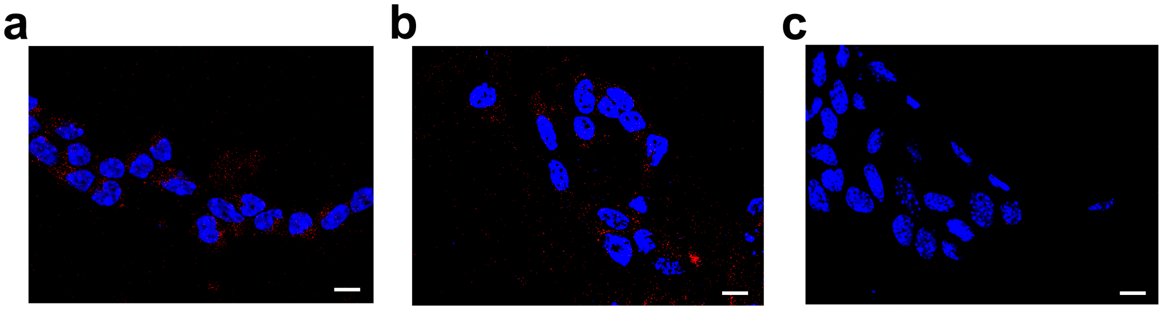

Supplementary Fig. 7 Receptor-AC5 complexes in striatal neurons in culture. Proximity Ligation assay (PLA) in striatal neurons in culture using antibodies against $A_{2A}R$ and AC5 (**a**), D_2R and AC5 (**b**) or only AC5, as negative control (**c**); confocal microscopy images (superimposed sections) shows $A_{2A}R$-AC5 or D_2R-AC5 complexes as red spots; cell nuclei were stained with DAPI (blue); scale bars: 5 µm.

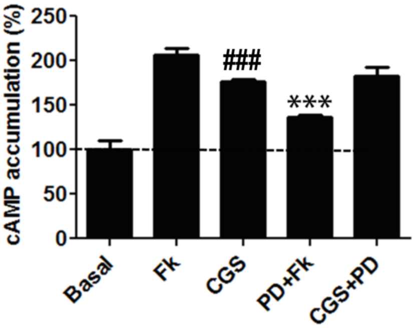

Supplementary Fig. 8 Lack of canonical Gs-Gi antagonistic interaction between $A_{2A}R$ and D_4R in striatal neurons in culture. cAMP production determined in rat striatal primary cultures exposed to CGS21680 (CGS; 100 nM), the D4R agonist PD168,077 (PD; 100 nM) or both in the absence or in the presence of forskolin (Fk; 0.5 µM), respectively. Values (in means ± SEM) are expressed as percentage of cAMP accumulation in non-treated cells (basal) (n = 4-6, with triplicates); $^{###}$: $p < 0.001$, as compared to basal values; ***: $p < 0.001$ as compared to Fk); no significant differences were detected between cells treated with CGS plus PD treated compared to CGS alone (one-way ANOVA followed by Tukey's multiple comparison tests).

Supplementary Table 1 Prediction of the topology of the TMs of AC5 with current algorithms

	Uniprot	TOPCONS	TMHMM	TMMOD	Phobius	Hydropathy
**TM 1**	196-216 ↑		194-216 ↑	196-216 ↑	197-216 ↑	1.676
**TM 2**	242-262 ↓	241 – 261 ↑	237-259 ↓	241-261 ↓	237-262 ↓	2.567
**TM 3**	268-288 ↑	268 – 288 ↓	269-287 ↑	268-288 ↑	268-288 ↑	2.614
**TM 4**	299-319 ↓	299 – 319 ↑	299-318 ↓	299-319 ↓	300-319 ↓	2.495
**TM 5**	325-345 ↑	326 – 346 ↓	328-345 ↑	328-345 ↑	325-343 ↑	**1.148**
**TM 5b**		348 – 368 ↑			350-367 ↓	2.194
**TM 6**	374-394 ↓	379 – 399 ↓			379-400 ↑	1.524
**TM 7**	770- 90 ↑	763 – 783 ↑	761-783 ↓	761-783 ↓	762-783 ↓	2.276
**TM 8**	792-812 ↓	789 – 809 ↓	787-809 ↑	790-810 ↑	788-813 ↑	2.119
**TM 9**	836-856 ↑	833 – 853 ↑	834-856 ↓	835-855 ↓	834-855 ↓	2.186
**TM 10**	910-930 ↓	909 – 929 ↓	909-931 ↑	910-930 ↑	909-927 ↑	1.910
**TM 11**	935-955 ↑	933 – 953 ↑	933-955 ↓	935-955 ↓	934-955 ↓	2.050
**TM 12**	984-1004 ↓	985 – 1005 ↓	984-1003 ↑	984-1004 ↑	984-1003 ↑	2.124

All algorithms predict the same 6 TMs for the M2 domain, but there is discrepancy on the predicted TMs of the M1 domain. According to TOPCONS (http://topcons.cbr.su.se), the first transmembrane helix corresponds to the same sequence predicted by the other algorithms for TM 2. Thus, another transmembrane helix (TM 1) is predicted more proximal to the AC5 N-terminus according to Uniprot (http://www.uniprot.org), TMHMM (http://www.cbs.dtu.dk/services/TMHMM), TMMOD (http://liao.cis.udel.edu/website/servers/TMMOD/scripts/frame.php?p=submit) and Phobius (http://phobius.sbc.su.se) algorithms. All algorithms predict the same TM 2 to TM 5, which correspond to the first four TMs according to TOPCONS. Two more TMs (TM 5b and TM 6) are then predicted according to TOPCONS and Phobius before the M2 domain and the TM 6 is also predicted by Uniprot. On the other hand, TM 5b and TM 6 are missing from TMHMM and TMMOD predictions, which leave M1 with only 5 TMs, while Phobius predicts a total of 7 TMs for the M1 domain. Outward and inward orientations are represented by upward and downward arrows, respectively. The last column indicates the average of Kyte-Doolittle hydropathy values of the putative TMs.

Supplementary Table 2 Amino acid sequence of TAT-TM peptides

A2AR-TM1	V^{8}YITVELAIAVLAILGNVLVCWAVW^{32}YGRKKRRQRRR
A2AR-TM2	YGRKKRRQRRRY^{43}FVVSLAAADIAVGVLAIPFAITI^{66}
A2AR-TM3	L^{78}FIACFVLVLTQSSIFSLLAIAI^{100}YGRKKRRQRRR
A2AR-TM4	YGRKKRRQRRRA^{121}KGIIAICWVLSFAIGLTPMLGW^{143}
A2AR-TM5	M^{174}NYMVYFNFFACVLVPLLLMLGVYL^{198}YGRKKRRQRRR
A2AR-TM6	YGRKKRRQRRRL^{235}AIIVGLFALCWLPLHIINCFTFF^{258}
A2AR-TM7	L^{267}WLMYLAIVLSHTNSVVNPFIYAY^{290}YGRKKRRQRRR
D2R-TM1	A^{38}TLLTLLIAVIVFGNVLVCMAVS^{60}YGRKKRRQRRR
D2R-TM2	YGRKKRRQRRRY^{71}LIVSLAVADLLVATLVMPWVVY^{93}
D2R-TM3	I^{109}FVTLDVMMCTASILNLCAISI^{130}YGRKKRRQRRR
D2R-TM4	YGRKKRRQRRRV^{152}TVMISIVWVLSFTISCPLLF^{172}
D2R-TM5	F^{189}VVYSSIVSFYVPFIVTLLVYIKIY^{213}YGRKKRRQRRR
D2R-TM6	YGRKKRRQRRRM^{374}LAIVLGVFIICWLPFFITHIL^{395}
D2R-TM7	A^{410}FTWLGYVNSAVNPIIYTTFNI^{431}YGRKKRRQRRR
AC5-TM1	YGRKKRRQRRRG^{196}AGPGAVLSLGACCLALLQIF^{216}
AC5-TM2	L^{242}TMLMAVLVLVCLVMLAFHAA^{262}YGRKKRRQRRR
AC5-TM2n	YGRKKRRQRRRL^{242}TMLMAVLVLVCLVMLAFHAA^{262}
AC5-TM3	YGRKKRRQRRRL^{268}PYLAVLAAAVGVILIMAVLC^{288}
AC5-TM3n	L268PYLAVLAAAVGVILIMAVLC^{288}YGRKKRRQRRR
AC5-TM4	G^{299}LACYALIAVVLAVQVVGLLL^{319}YGRKKRRQRRR
AC5-TM4n	YGRKKRRQRRRG^{299}LACYALIAVVLAVQVVGLLL^{319}
AC5-TM5	YGRKKRRQRRRA^{325}SEGIWWTVFFIYTIYTLLPV^{345}
AC5-TM5n	A^{325}SEGIWWTVFFIYTIYTLLPV^{345}YGRKKRRQRRR
AC5-TM5s	YGRKKRRQRRRLFATVITEVGSIPLFYWWIYT
AC5-TM5b	YGRKKRRQRRRA^{349}AVLSGVLLSALHLAIAL^{366}
AC5-TM6	F^{374}LLKQLVSNVLIFSCTNIVGV^{394}YGRKKRRQRRR
AC5-TM6n	YGRKKRRQRRRF^{374}LLKQLVSNVLIFSCTNIVGV^{394}
AC5-TM7	YGRKKRRQRRRL^{770}VFLFICFVQITIVPHSIFML^{790}
AC5-TM8	F^{792}YLTCSLLLTLVVFVSVIYSC^{812}YGRKKRRQRRR
AC5-TM9	YGRKKRRQRRRL^{836}VGVFTITLVFLAAFVNMFTC^{456}
AC5-TM10	F^{910}TYSVLLSLLACSVFLQISCI^{930}YGRKKRRQRRR
AC5-TM11	YGRKKRRQRRRL^{935}MLAIELIYVLIVEVPGVTLF^{955}
AC5-TM12	V^{984}ALKVVTPIIISVFVLALYLH^{1004}YGRKKRRQRRR

References

1. Pronk, S. et al. GROMACS 4.5: a high-throughput and highly parallel open source molecular simulation toolkit. *Bioinformatics* **29,** 845-854 (2013).
2. Cordomí, A., Caltabiano, G. & Pardo, L. Membrane Protein Simulations Using AMBER Force Field and Berger Lipid Parameters. *J. Chem. Theory Comput.* **8,** 948-958 (2012).
3. Martí-Renom, M. A. et al. Comparative protein structure modeling of genes and genomes. *Annu. Rev. Biophys. Biomol. Struct.* **29,** 291-325 (2000).
4. Rasmussen, S. G. et al. Crystal structure of the β2 adrenergic receptor-Gs protein complex. *Nature* **477,** 549-555 (2011).
5. Sadana, R., Dascal, N. & Dessauer, C. W. N terminus of type 5 adenylyl cyclase scaffolds Gs heterotrimer. *Mol. Pharmacol.* **76,** 1256-1264 (2009).
6. Xie, K. et al. Stable G protein-effector complexes in striatal neurons: mechanism of assembly and role in neurotransmitter signaling. *Elife* **4,** e10451 (2015).
7. Tesmer, J. J., Sunahara R. K., Gilman, A. G. & Sprang, S. R. Crystal structure of the catalytic domains of adenylyl cyclase in a complex with Gsalpha-GTPgammaS. *Science* **278,** 1907-1916 (1997).
